# Factors Influencing Quality of Life in Myasthenia Gravis Patients: Results From a Cross‐Sectional Study in South of Iran

**DOI:** 10.1002/brb3.70680

**Published:** 2025-08-21

**Authors:** Fatemeh Khodakarami, Arashk Mallahzadeh, Mohammad Fararouei, Abbas Rahimi Jaberi, Maryam Owjfard

**Affiliations:** ^1^ Clinical Neurology Research Center Shiraz University of Medical Sciences Shiraz Iran; ^2^ Department of Epidemiology, HIV/AIDS Research Center, School of Health Shiraz University of Medical Sciences Shiraz Iran

**Keywords:** Activities of Daily Living (ADL), depression, fatigue, myasthenia gravis, quality of life

## Abstract

**Background:**

Myasthenia gravis (MG) is an autoimmune disorder of the neuromuscular junction characterized by fatigue and muscle weakness, often leading to significant impairments in quality of life (QOL). This single‐center cross‐sectional study investigated factors influencing QOL among MG patients.

**Methods:**

Fifty‐two participants diagnosed with MG were assessed using the MG Foundation of America (MGFA) classification and the MG composite (MGC) scale for disease severity. Patients completed validated questionnaires, including the Fatigue Severity Scale (FSS), Beck Depression Inventory (BDI), MG Activities of Daily Living (MG‐ADL), and the MG Quality of Life 15‐item scale (MG‐QOL). Family stress levels were self‐reported and categorized as “Rarely/Never,” “Sometimes,” “Often,” or “Always.”

**Results:**

The analysis revealed no significant differences in QOL scores based on sex, disease type, presenting symptoms, thymectomy status, or BMI. Employed or freelance patients exhibited significantly lower QOL and higher fatigue compared to retired or housewife groups. Higher family stress correlated with increased fatigue, worse MGC scores, and poorer QOL. Regression analysis identified the MG‐ADL and FSS scores as the strongest predictors of QOL.

**Conclusion:**

QOL in MG is influenced by complex interactions among functional, social, and occupational factors. These findings emphasize the need for tailored interventions to address fatigue and support occupational and familial well‐being in MG patients. Further research with larger cohorts is warranted to refine these insights and guide clinical strategies.

## Graphical abstract

Fatigue, occupational status, and family stress significantly impact quality of life in myasthenia gravis patients, with fatigue and functional ability being the strongest predictors, highlighting the need for tailored interventions to improve patient well‐being.

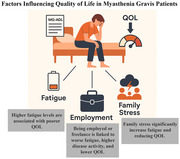

## 1 Introduction

Myasthenia gravis (MG) is a chronic autoimmune disorder of the neuromuscular junction characterized by skeletal muscle weakness and fluctuating fatigue of the frequently used muscles (Carr et al. 2010). Although MG is considered treatable, its progressive nature and variable symptomatology contribute to significant morbidity and a rising global prevalence over recent decades (Dresser et al. 2021). The condition profoundly impacts health‐related quality of life (QOL), with studies demonstrating that MG impairs physical functioning, mental health, and social participation of patients (Gelinas et al. 2022).

Approximately 85% of MG patients possess autoantibodies targeting the acetylcholine receptor (AChRAb), whereas others exhibit antibodies against muscle‐specific kinase (MuSKAb) or lipoprotein‐related protein 4 (LRP4) (Lindstrom et al. 1998; Hoch et al. 2001; Zisimopoulou et al. 2014). Thymic abnormalities, including thymomas or hyperplasia, are closely linked to MG pathogenesis and may exacerbate QOL deficits by prolonging disease severity or necessitating invasive interventions (Dresser et al. 2021). Notably, women in MG cohorts (who have higher rates of thymoma) have shown significantly worse MG‐specific QOL scores than men, although this sex difference was eliminated by thymectomy (Lee et al. 2018).

Managing MG typically involves long‐term therapies tailored to symptom severity and thymic involvement, including acetylcholinesterase inhibitors, immunosuppressive medications, plasma exchange, intravenous immunoglobulin, or thymectomy (Sanders et al. 2016; Wolfe et al. 2016). Although patients undergoing thymectomy demonstrate significant clinical improvement, their QOL does not differ significantly compared to those who do not undergo the procedure (Wolfe et al. 2016; Kanikannan et al. 2022). Immunosuppressants like corticosteroids can reduce relapse rates but are associated with metabolic and psychological side effects that further burden patients and impact their QOL (Utsugisawa et al. 2020). For example, large surveys in Australia found that the majority (57%–85%) of MG patients experience treatment‐related side effects (fatigue, weight gain, infection risk, etc.) that substantially burden daily function and QOL (Sansoni et al. 2023). Conversely, they found that patients who had undergone thymectomy reported better overall QOL than those who did not. Specifically, worse disease severity and disability (higher MGFA class or MG‐ADL score) have been consistently linked to poorer QOL in MG (Wu et al. 2023). Psychological comorbidities such as depression and anxiety independently predict further QOL impairment (Alanazy et al. 2020).

Given the multifaceted nature of QOL impairment in MG and the influence of social, psychological, and occupational dynamics, region‐specific insights are essential for guiding tailored care strategies. This study was conducted to identify the key clinical and psychosocial determinants of QOL in MG patients in Southern Iran, to support more effective and patient‐centered treatment strategies.

## 2 Materials and Methods

### 2.1 Study Population

This cross‐sectional study examined all MG patients who attended the educational hospitals and medical centers affiliated with Shiraz University of Medical Sciences between August 2022 and March 2023. This study was approved by the research ethics committee of Shiraz University of Medical Sciences (Ethical code: IR.SUMS.MED.REC.1401.044).

Inclusion criteria required a confirmed diagnosis of MG by a neurologist, age >18 years, available serum antibody tests, and willingness to participate. Exclusion criteria were a known psychiatric disorder or a personal or family history of any mental illness. Of the registered patients, 15 were excluded on the basis of these criteria, leaving a final sample of 52 MG patients. Following written informed consent, participants underwent a comprehensive evaluation by a neurologist and were classified according to the MG Foundation of America (MGFA) criteria and disease severity using the MG composite (MGC) scale (Burns et al. 2010). We stratified patients into three different age groups at the time of diagnosis for analysis and comparison. Under 25 years old, 25 to 45 years old, and over 45 years old.

### 2.2 Questionnaires

Questionnaires regarding demographic characteristics, weight, Fatigue Severity Scale (FSS) questionnaire, Activities of Daily Living (ADL) questionnaire, MG‐QOL questionnaire, and Beck Depression Inventory (BDI) questionnaire were completed by the patients alone or with assistance as required. All questionnaires were administered as standardized paper forms during clinical visits under researcher supervision.

**The FSS** is a reliable and valid tool useful for detecting important aspects of MG‐related fatigue in clinical settings. It is a self‐assessment tool, first introduced in 1989, primarily for the benefit of patients suffering from multiple sclerosis (Krupp et al. 1989). It's important to note that this scale doesn't specifically evaluate cognitive fatigue. The FSS comprises nine concise items, each rated on a scale from 1, indicating strong disagreement, to 7, indicating strong agreement, with higher scores reflecting greater levels of fatigue. Reliability and validity of the Persian version of the FSS have been confirmed (Fereshtehnejad et al. 2013).

**The BDI questionnaire** is a reliable and valid questionnaire to examine the following components of feelings and behaviors: low spirits, negative outlook, feelings of inadequacy, absence of contentment, remorse, self‐loathing, self‐injury, isolation from society, hesitation, skewed self‐perception, challenges at work, exhaustion, and diminished hunger (Beck and Beck 1972). The scoring is as follows: normal (0–3); slight depression (4–7); mild‐to‐moderate depression (8–11); moderate depression (12–15); and severe depression (16–39). All the items had been validated in Persian (Vasegh and Baradaran 2014).

**The MG**‐**ADL** questionnaire is a reliable and valid 8‐item scale reported by the patient that measures MG symptoms and the patient's functional status, and it was used in our study to assess individual daily activities. This tool is made up of eight components: Two evaluate eye‐related issues, 3 target throat‐related problems, 1 focuses on breathing difficulties, and 2 are concerned with limb‐related disorders. Each component is assigned a value from 0 to 3, resulting in a total possible score ranging from 0 to 24. An increased total indicates a deteriorating health status of the individual. Reliability and validity of the Persian version of the ADL questionnaire have been confirmed (Faghani et al. 2022).

**The** MG‐QOL is a health‐related QOL assessment tool specifically for patients with MG that is easily administered and interpreted. Validation studies of MG‐QOL have shown its structural validity, and it is a useful and valuable tool for evaluating patients with MG in Persian (Ostovan et al. 2016). The MG‐QOL self‐reported tool has been very useful in informing physicians about the patient's perception and tolerance of MG. This measure is composed of 15 components that explore aspects related to well‐being in terms of health. Each component is assigned a value from 0 to 4, leading to an overall possible value that spans from 0 to 60. An elevated overall value signifies a deteriorating state of well‐being for the individuals.

Additionally, family stress was evaluated via researcher‐administered verbal interview using the question, “How frequently do you experience family‐related conflict or stress?” with four response options (Never/Rarely/Sometimes/Often).

### 2.3 Statistical Analysis

Data were collected and analyzed using SPSS software (Version 23 for Windows). The Shapiro–Wilk test was performed to determine normality of distribution. Qualitative variables are presented as percentage (%) and the results for quantitative variables are presented as median and IQR due to skewed variables. For subgroup analysis of the MG‐BDI, MG‐ADL, MG‐FSS, MGC, and MG‐QOL scales, Mann–Whitney *U*‐test was used for analysis between two subgroups and the Kruskal–Wallis test was used for analysis between more than two subgroups with post hoc multiple comparisons as appropriate with the Bonferroni correction applied manually. Subgroups for comparison included gender, age group, disease type, disease presentation, Thymectomy, Occupation, BMI, MGFA class, and stress in family. Spearman correlation analysis was performed to test for correlation between variables. Multiple linear regression analysis was performed with the dependent variable being MG‐QOL and the method enter was employed in which all selected independent variables were entered into the model simultaneously. A *p* value below 0.05 was considered statistically significant.

## 3 Results

The demographic and clinical characteristics of the 52 included patients are summarized in Table 1. Males comprised 32.7% of our sample and females 67.3%. Regarding age at diagnosis, 19.2% were below 25, 51.9% were 25–44, and 28.8% were above 44 years old. Overall, 71.2% of patients were anti‐Ach+, and 28.8% were either Anti‐Musk or seronegative.

**TABLE 1:** Demographics and clinical manifestation of included participants.

Characteristic	*N*, %	Characteristic	*N*, %
**Age at diagnosis (years)**		**Presenting symptom**	
<25	10 (19.2)	Ocular	37 (71.2)
25–44	27 (51.9)	Bulbar/General weakness	15 (28.8)
>44	15 (28.8)		
**Gender**		**Thymectomy**	
Male	17 (32.7)	Yes	21 (40.4)
Female	35 (67.3)	No	31 (59.6)
**Marital status**		**DMT**	
Single	14 (26.9)	Mestinon	32 (61.5)
Married	38 (73.1)	Prednisolone	31 (59.6)
**Education level**		Rituximab	7 (13.5)
Elementary	16 (30.8)	Corticosteroid‐sparing	9 (17.3)
Middle/High school	20 (38.5)	IVIG	2 (3.8)
Beyond college	16 (30.8)		
**Occupation**		**Stress in family**	
Housewife/Retired	39 (75)	Never/Rarely	11 (21.2)
Freelance/Employee	13 (25)	Sometimes	15 (28.8)
**BMI**		Often	13 (25)
Normal/Underweight	24 (46.2)	Always	13 (25)
Overweight	15 (28.8)		
Obese	13 (25)		
**Disease type**			
Anti‐AChR+	35 (71.2)		
Seronegative/Anti‐MuSK+	17 (28.8)		

Abbreviation: DMT, disease‐modifying therapy.

The median and IQR for the MGC, MG‐ADL, MG‐BDI, MG‐FSS, and MG‐QOL questionnaires are presented in Table 2.

**TABLE 2:** Median and IQR scores of the questionnaires utilized in the study.

Variable	Value (median, IQR/frequency %)
MGC	1, 0–6
BDI	13, 3.25–23.75 23 normal (44.2%) 14 mild mood disturbance/borderline depression (26.9%) 7 moderate depression (13.5%) 8 Severe depression (15.4%)
Average FSS	5.44, 4.03–6.9 43 problematic fatigue (82.7%)
Total ADL	2, 0–6.75
Total QOL	17, 8–40.75
MGFA	21 Remission (40.4%) 4 Class I (7.7%) 16 Class II (30.8%) 8 Class III (15.4%) 3 Class IV (5.8%)

Abbreviations: ADL, Activities of Daily Living; BDI, Beck Depression Inventory; FSS, Fatigue Severity Scale; MGC, Myasthenia Gravis composite; MGFA, MG Foundation of America; QOL, quality of life.

Subgroup analysis indicated no significant differences in any questionnaire scores between males and females. Similarly, no statistically significant differences were observed across subgroups based on disease type, presenting symptoms, thymectomy status, and BMI (Table 3).

**TABLE 3:** Subgroup analysis for comparison between Myasthenia Gravis Activities of Daily Living (MG‐ADL), Beck Depression Inventory (BDI), Fatigue Severity Scale (FSS), MG composite (MGC), and MG Quality of Life 15‐item scale (MG‐QO) (Median, IQR).

		MG‐ADL	BDI	FSS	MGC	MG‐QOL
Sex	Female	2, 0–5	13, 5–23	4.88, 3.11–6.88	1, 0–5	16, 8–32
	Male	6, 0–8.5	13, 1.5–26	6.33, 4.55–7	4, 0–11	35, 10–43.5
	*p* value	0.18	0.87	0.13	0.32	0.16
Disease type	Anti‐Ach+	2, 0–8	11, 4–24	5.22, 3.11–6.88	1, 0–8	16, 6–33
	Anti‐Musk+/Seronegative	2, 0–6.5	14, 1–21.5	5.88, 4.38–6.88	1, 0–5	21, 11–42
	*p* value	0.74	0.96	0.42	0.74	0.40
Age groups	Age <25	2, 0.75–9.25	9, 1.5–31	4.61, 4.27–6.5	3, 0–10.25	12, 7.5–44.75
	25–45	2, 0–6	13, 4–23	5.44, 3.11–6.55	2, 0–8	17, 8–33
	Age >45	2, 0–7	15, 4–27	5.88, 4–7	0, 0–1	21, 8–44
	*p* value	0.88	0.72	0.75	0.031^*^	0.76
Disease presenting sign	Ocular	2, 0–7	13, 3.5–24	5.77, 3.05–6.88	1, 0–5.5	20, 7–42
	Bulbar or weakness	1, 0–7	9, 2–23	4.77, 4.11–7	2, 0–8	14, 8–35
	*p* value	0.48	0.49	0.95	0.89	0.64
Thymectomy	Yes	1, 0–6	12, 3.5–24.5	4.77, 3.77–6.38	2, 0–6.5	14, 7–30
	No	3, 0–7	13, 3–20	5.44, 4–7	1, 0–6	20, 8–43
	*p* value	0.14	0.95	0.51	0.96	0.41
Occupation	Employee/Freelance	6, 2–9	10, 5–22	6.88, 5.88–7	6, 0–13.5	35, 16–43
	Housewife/Retired	2, 0–5	13, 3–24	4.77, 3.11–6.33	1, 0–4	14, 8–32
	*p* value	0.021^*^	0.96	0.004^**^	0.20	0.045^*^
BMI	Normal or below‐normal	3, 0.25–8	14.5, 5.25–26.5	5.44, 3.02–6.41	3, 0–6	18.5, 8.25–42.25
	Overweight	1, 0–5	10, 1–16	4.33, 3.11–6.88	0, 0–4	14, 4–27
	Obese range or higher	2, 0–5	12, 1–23	5.88, 4.38–7	1, 0–8	17, 12.5–45.5
	*p* value	0.30	0.42	0.26	0.24	0.23
MGFA class	Remission	0, 0–2	9, 2.5–20	4.11, 2–6.94	0	12, 3.5–20.5
	Class I	0, 0–4.5	19, 2–36	4.33, 4.08–5.5	1, 0.25–1	12.5, 5–40.25
	Class II	3, 1.25–7.5	13.5, 6–16.75	5.72, 4.52–6.5	3, 2–5.75	16.6, 10.5–40
	Class III	7.5, 2.75–11.5	18, 9.5–31.5	6.72, 5.5–7	8.5, 6–25.75	33.5, 20.25–49.75
	Class IV	10, 4–10	34, 2–34	6.33, 5.77–6.33	20, 14–20	50, 33–50
	*p* value	<0.000^**^	0.53	0.63	<0.000^**^	0.025^*^
Stress in family	Never/Rarely	0, 0–3	6, 2–13	3.44, 1.44–4.33	0, 0–1	5, 3–13
	Sometimes	2, 0–8	11, 3–25	5.44, 4.11–6.33	2, 0–9	15, 12–43
	Often	6, 1.5–8.5	15, 9.5–20.5	6.33, 5.5–7	6, 1.5–12.5	33, 18.5–40.5
	Always	2, 0–8.5	20, 6–36	5.88, 3.61–6.94	0, 0–2	20, 6–44
	*p* value	0.10	0.09	0.018^*^	0.025^*^	0.006^*^

* *p* < 0.05.

** *p* < 0.005.

Patients diagnosed at the age of 25–44 showed higher MGC scores than those diagnosed after 45 (2, 0, respectively, *p* = 0.012).

Our results show that patients who were employees or freelancers demonstrated higher MG‐ADL scores compared to those who were retired or housewives (6, 2, respectively, *p* = 0.021). Occupied patients also showed higher FSS scores compared to retired or housewife patients (6.88, 4.77, respectively, *p* = 0.004). QOL was significantly worse in employed and freelance patients compared to non‐employed individuals (35, 14, respectively, *p* = 0.045).

MG‐ADL scores were significantly lower in patients under remission (0) compared with patients in the Class II (3) (*p* = 0.004), Class III (7.5) (*p* = 0.001), and Class IV (10) (*p* = 0.004) MGFA categories. Although a significant difference was observed in QOL among different MGFA class patients, these differences did not reach significance after Bonferroni correction.

Patients often experiencing conflict and stress in family showed higher FSS scores compared to those never/rarely experiencing family stress (6.33, 3.44, respectively, *p* = 0.002). Similarly, they demonstrated higher MGC scores (6, 0, respectively, *p* = 0.006). QOL was significantly worse in the group reporting never/rarely experiencing stress in family compared to those who reported often experiencing such events (33, 5, respectively, *p* < 0.001).

The Spearman correlation analysis revealed that MG‐QOL score was significantly correlated with MGC (*R* = 0.506, *p* < 0.001), BDI (*R* = 0.619, *p* < 0.001), FSS (*R* = 0.811, *p* < 0.001), and ADL (*R* = 0.783, *p* < 0.001). Multivariate linear regression analysis was performed to identify independent predictors of QOL using age at diagnosis, sex, occupation, family stress, MGC, BDI, FSS, and ADL as independent variables. The model was statistically significant (*F*(8, *X*) = 17.299, *p* < 0.001), explaining 76.3% of the variance in QOL (*R* ^2^ = 0.763, Adjusted *R* ^2^ = 0.719). Only FSS and ADL were significant predictors. A significant positive association was observed between FSS scores and QOL (*β* = 0.385, *p* = 0.001), where higher fatigue levels correlated with poorer QOL. ADL was the strongest predictor (*β* = 0.566, *p* < 0.001). Other predictors, including age, sex, occupation, family stress, MGC, and BDI, were not significant.

## 4 Discussion

In the present study, we aimed to identify significant predictors of QOL among patients living with MG. Due to the wide range of MG symptoms and its effect on various physical and mental domains, MG affects QOL on many levels (Jayam Trouth et al. 2012; Szczudlik et al. 2020).

Although earlier studies have reported lower QOL in patients with generalized symptoms compared to those with purely ocular symptoms (Rostedt et al. 2006), our findings revealed no significant difference in QOL based on symptom presentation. Similarly, thymectomy status did not significantly influence QOL, aligning with prior research (Szczudlik et al. 2020). These results suggest that the effects of symptom type presentation and surgical intervention on QOL may vary across populations. The impact of thymectomy on a patient's QOL is a reflection on how the procedure has decreased the patients’ symptoms, and future studies should consider this matter.

Regarding age, existing literature shows mixed results. Some studies have noted lower QOL in older patients due to reduced physical functioning (Jeong et al. 2018). Another study highlighted worse QOL in late‐onset MG patients across both physical and mental domains (Szczudlik et al. 2020). However, in our study, age at diagnosis was not a significant determinant of QOL. This discrepancy underscores the variability in findings, likely influenced by the heterogeneity of assessment tools and patient populations. Distinguishing between one's physical and mental QOL is crucial to further investigate the effects of age on QOL.

Our findings highlight the complex relationship among occupational status, fatigue, and QOL in patients with MG. Patients who were employed or working as freelancers demonstrated significantly higher ADL scores and lower QOL compared to those who were retired or housewives. Although previous studies have found that the lack of employment is linked to lower QOL in MG patients (Szczudlik et al. 2020), many factors need to be addressed, such as job type, workload, and workplace accommodations (Harris et al. 2019). Occupation‐related issues are a neglected matter among MG patients (Guastafierro et al. 2020). MG mainly affects individuals in working age and causes significant limitations in work duties and employment status (Paul et al. 2001). Progressive physical limitations in MG patients often necessitate workplace adaptations, including reduced work hours or transition to less physically demanding roles (Paul et al. 2001). A meta‐analysis demonstrated that MG is responsible for a 15%–35% decrease in the number of employed individuals (Guastafierro et al. 2020). These employment disruptions not only diminish QOL but may also create financial barriers to optimal care (Guastafierro et al. 2020). Systematic evaluation of MG's occupational consequences represents an important avenue for future research.

Interestingly, patients reporting frequent family stress/conflict demonstrated higher fatigue and MGC scores, and their QOL scores were higher, which indicates poorer QOL, than those with minimal or no family stress. These results underscore the complex psychosocial factors that influence QOL and highlight the importance of addressing family dynamics in QOL assessment.

BMI and obesity are significant determinants of QOL not only in certain diseases but also in the general population (Fontaine and Barofsky 2001). Among our sample population, 25% were obese and 28.8% were overweight. This may be due to reduced physical activity in these individuals. When evaluating the impact of BMI on QOL, it is important to differentiate between the physical and mental components, as BMI is more likely to influence physical well‐being. Studies have shown that higher BMI is associated with poorer physical QOL while having little to no effect on the mental component (Szczudlik et al. 2020; Winter et al. 2010). Another study conducted in Japan using MG‐QOL15 found that BMI was not related to lower QOL (Utsugisawa et al. 2014). Furthermore, in the current study, we did not find a significant relation between BMI and QOL. These results suggest that findings differ on the basis of the scales used to measure QOL, and BMI most likely affects the physical aspects of QOL rather than mental subscales. Future studies with adequate sample size and adjustments are required to address this.

Higher MGFA scores have been shown to be related to lower QOL in MG patients. Szczudlik et al. (2020) found that MGFA was related to general health as well as physical and mental components of QOL. Another study also found that worsening of disease status based on the MGFA class is linked to lower QOL (Kumar et al. 2016). However, our findings differ, as we found that QOL did not differ significantly among MGFA groups. This divergence may reflect variations in disease perception, patient resilience, or limitations of cross‐sectional analyses in capturing longitudinal disease impact.

Our results indicate that ADL and FSS were the most dominant predictors of QOL. Studies have shown the burden of fatigue in MG and its relation to reduced QOL (Hoffmann et al. 2016). Fatigue can be seen independent from muscle weakness and should not be overlooked by healthcare providers (Akkan Suzan et al. 2022). These findings highlight the importance of addressing fatigue and enhancing daily functioning in MG management to improve patients’ overall QOL.

Although we have provided evidence in the current study on the role of fatigue and ADL in QOL, our study comes with limitations. First, the small sample size reduces the generalizability of our findings. We did not include a control group, and the cross‐sectional design prevents us from establishing cause‐and‐effect relationships. We did not distinguish between physical and mental aspects of QOL. The small sample size of non‐AChR+ MG patients (Anti‐MuSK, seronegative) prevented meaningful separate analyses of these subgroups. Future studies should recruit larger samples of Anti‐MuSK+ and seronegative patients to investigate differences in QOL.

Moreover, recall bias might exist in reporting the questionnaires. Self‐reporting of family stress and conflict is another limitation. Although the effect size was substantial, the relatively small sample size in relation to the number of predictors may limit the robustness and generalizability of the results. Lastly, we did not take into account medical or surgical comorbidities, which may impact QOL.

Future studies with large sample sizes including diverse populations would allow us to better understand how factors like fatigue and ADL influence QOL and to develop more effective, targeted interventions.

## 5 Conclusion

QOL in individuals living with MG is influenced by multiple interacting factors. In this study, higher fatigue levels (FSS) and greater limitations in daily activities (MG‐ADL) were significantly associated with poorer QOL, with both emerging as the strongest independent predictors. Patients who were employed or self‐employed reported worse QOL, higher fatigue, and greater functional limitations compared to those who were retired or homemakers. Frequent family stress was also linked to increased fatigue, more severe disease symptoms, and lower QOL. These findings underscore the negative impact of physical, occupational, and psychosocial burdens on QOL in MG. Future longitudinal studies are needed to confirm these associations and develop interventions tailored to reduce fatigue, improve functional ability, and support social and emotional well‐being in this population.

## Author Contributions

**Fatemeh Khodakarami**: methodology, formal analysis, investigation. **Arashk Mallahzadeh**: methodology, formal analysis, investigation, writing – original draft, writing – review and editing. **Mohammad Fararouei**: methodology, writing – review and editing. **Abbas Rahimi Jaberi**: conceptualization, writing – review and editing, supervision. **Maryam Owjfard**: conceptualization, writing – original draft.

## Conflicts of Interest

The authors declare no conflicts of interest.

## Peer Review

The peer review history for this article is available at https://publons.com/publon/10.1002/brb3.70680.

## Data Availability

Data supporting the findings of this study are available from the corresponding author upon reasonable request.
